# Construction of an MLR-QSAR Model Based on Dietary Flavonoids and Screening of Natural α-Glucosidase Inhibitors

**DOI:** 10.3390/foods11244046

**Published:** 2022-12-14

**Authors:** Ting Yang, Zichen Yang, Fei Pan, Yijia Jia, Shengbao Cai, Liang Zhao, Lei Zhao, Ou Wang, Chengtao Wang

**Affiliations:** 1 Beijing Engineering and Technology Research Center of Food Additives, Beijing Technology and Business University, Beijing 100048, China; 2 Institute of Apicultural Research, Chinese Academy of Agricultural Sciences, Beijing 100093, China; 3 Yunnan Institute of Food Safety, Kunming University of Science and Technology, Kunming 650500, China; 4 National Institute for Nutrition and Health, Chinese Center for Disease Control and Prevention, Beijing 100050, China

**Keywords:** dietary flavonoids, α-glucosidase, molecular fingerprint similarity, MLR-QSAR, molecular dynamics simulation

## Abstract

Postprandial hyperglycemia can be reduced by inhibiting α-glucosidase activity. Common α-glucosidase inhibitors such as acarbose may have various side effects. Therefore, it is important to find natural products that are non-toxic and have high α-glucosidase-inhibitory activity. In the present study, a comprehensive computational analysis of 27 dietary flavonoid compounds with α-glucosidase-inhibitory activity was performed. These included flavonoids, flavanones, isoflavonoids, dihydrochalcone, flavan-3-ols, and anthocyanins. Firstly, molecular fingerprint similarity clustering analysis was performed on the target molecules. Secondly, multiple linear regression quantitative structure–activity relationship (MLR-QSAR) models of dietary flavonoids (2D descriptors and 3D descriptors optimized), with R^2^ of 0.927 and 0.934, respectively, were constructed using genetic algorithms. Finally, the MolNatSim tool based on the COCONUT database was used to match the similarity of each flavonoid in this study, and to sequentially perform molecular enrichment, similarity screening, and QSAR prediction. After screening, five kinds of natural product molecule (2-(3,5-dihydroxyphenyl)-5,7-dihydroxy-4H-chromen-4-one, norartocarpetin, 2-(2,5-dihydroxyphenyl)-5,7-dihydroxy-4H-chromen-4-one, 2-(3,4-dihydroxyphenyl)-5-hydroxy-4H-chromen-4-one, and morelosin) were finally obtained. Their IC_50pre_ values were 8.977, 31.949, 78.566, 87.87, and 94.136 µM, respectively. Pharmacokinetic predictions evaluated the properties of the new natural products, such as bioavailability and toxicity. Molecular docking analysis revealed the interaction of candidate novel natural flavonoid compounds with the amino acid residues of α-glucosidase. Molecular dynamics (MD) simulations and molecular mechanics/generalized Born surface area (MMGBSA) further validated the stability of these novel natural compounds bound to α-glucosidase. The present findings may provide new directions in the search for novel natural α-glucosidase inhibitors.

## 1. Introduction

Diabetes mellitus is a metabolic disorder characterized by chronic hyperglycemia caused by various etiologies. Diabetes can be divided into different types according to its etiology. The common clinical types of diabetes are type 1 diabetes, type 2 diabetes, and gestational diabetes. Type 2 diabetes mellitus (T2DM) is the main type of diabetes, accounting for 90–95% of the incidence of diabetes [1]. T2DM can lead to certain secondary complications, such as nerve damage, kidney failure, and blindness [2]. Controlling postprandial blood glucose plays an important role in interfering with T2DM. Inhibiting the activity of α-glucosidase can slow down or inhibit the digestion and absorption of carbohydrates, thereby reducing postprandial hyperglycemia [3].

α-Glucosidase, an enzyme that catalyzes the hydrolysis of glycosidic bonds in complex carbohydrates, is present in the intestinal brush border membrane. It catalyzes the hydrolysis of α-(1→4)-glycosidic bonds of sugars (disaccharides and starches), releasing free monosaccharides (α-D-glucose) in the final step of carbohydrate digestion [4]. The released monosaccharides are absorbed by the gut, resulting in increased blood sugar and insulin levels [5]. Therefore, α-glucosidase inhibitors can delay the release of α-D-glucose from complex dietary carbohydrates and delay the absorption of glucose, thereby reducing blood glucose levels and suppressing postprandial hyperglycemia [6].

α-Glucosidase inhibitors can inhibit the digestion and absorption of carbohydrates by reversibly competing with carbohydrate molecules for the binding site of α-glucosidase on the brush border of small intestinal epithelial cells, thereby reducing postprandial blood glucose [7]. α-Glucosidase inhibitors are a unique class of antidiabetic agents that have been described as attractive therapeutic targets for T2DM [8]. Therefore, the development of α-glucosidase inhibitors has become a hotspot in the current research. At present, typical clinical drugs for the treatment of non-insulin-dependent diabetes mellitus by this approach include acarbose, voglibose, miglitol, etc. [9]. Although these inhibitors have good effects in regulating blood sugar levels, they may have various side effects, such as diarrhea, flatulence, liver disease, and abdominal cramps [10]. Numerous studies have shown that natural products (e.g., active natural ingredients and crude extracts) can inhibit α-glucosidase activity with no or few side effects [11]. Therefore, more and more attention has been paid to finding α-glucosidase inhibitors with less toxicity or side effects from natural products.

Flavonoids are natural compounds that exist widely in various plants [12], many of which possess high biological activities, such as reducing vascular fragility, improving vascular permeability, reducing blood lipids and cholesterol, etc. [13]. The basic structure of flavonoids takes C6-C3-C6 as the nucleus, forming a series of sub-class compounds, mainly including flavones, flavanones, isoflavones, flavonols, flavanols, anthocyanins, and chalcones [14]. Studies have shown that many flavonoids can inhibit α-glucosidase activity and may have the potential to improve postprandial blood glucose or T2DM [15]. Although some studies have explored the structure–activity relationship between flavonoids and glucosidases [16,17,18], few studies have explored this structure–activity relationship at the molecular topological level. On the other hand, it is more important to use the optimized model application for the screening of natural anti-α-glucosidase flavonoids.

In recent years, quantitative structure–activity relationship (QSAR) models have been widely used for the quantitative analysis of the structure–activity relationships of compounds [19]. QSAR modeling based on computer assistance can be an important tool to guide the design and screening of new α-glucosidase inhibitors with higher activity, which can remarkably reduce the cost of discovery [20]. Therefore, in this study, QSAR models were established based on the previous experimental data [21]. We then used these models with our previously developed natural product clustering library tool, MolNatSim, to enrich and screen for natural anti-α-glucosidase flavonoids. Finally, ADMET prediction, molecular dynamics (MD) simulations, and molecular mechanics/generalized Born surface area (MMGBSA) were conducted to verify the potential pharmacokinetics of these candidate inhibitors and their binding stability to α-glucosidase.

## 2. Materials and Methods

### 2.1. Preparation of Datasets

The sample set for this study was derived from 27 dietary flavonoids with α-glucosidase-inhibitory activity from our previously measured data [21]. α-Glucosidase (from *Saccharomyces cerevisiae*, 58.55 units/mL, EC 3.2.1.20) and p-nitrophenyl-α-D-glucopyranoside were purchased from Sigma (Sigma-Aldrich, Shanghai, China). Flavonoid standards (purity ≥ 98.0%) were obtained from Chengdu Must Biotechnology Co., Ltd. (Chengdu, Sichuan, China). The inhibition of α-glucosidase activity was performed using a modified method with *p*-nitrophenyl-α-D-glucopyranoside (*p*-NPG) as a substrate. The reaction was started by adding 4 mmol L^−1^ *p*-NPG (diluted in 50 mmol L^−1^ phosphate buffer, pH 6.8). The reaction mixture was incubated at 37 °C for 10 min, and the absorbance values were measured at 405 nm using a Spectra Max M5 microplate meter. Acarbose (purity > 95%) was used as a positive control, and 5% DMSO was used as a negative control. These molecules were used to perform the QSAR analysis. The average IC_50_ values of flavonoids for α-glucosidase inhibition were converted to pIC_50_ (−log IC_50_) values as the dependent variable for QSAR analysis. The QSAR model was constructed by using MolAICal (version 1.3) [22] software. Additionally, the whole dataset was split into two datasets: 80% of the flavonoids were used as the training set, and the remaining 20% were used as the validation set, which was used to generate the final QSAR models and evaluate the predictive power of the derived models.

### 2.2. Structural Modeling

SMILES of dietary flavonoids were taken from the PubChem database, and the SMILES format for each dietary flavonoid was obtained using the RDKit program (version 2021.09, http://www.rdkit.org/ (accessed on 8 June 2022)) [23]. All structures performed energy minimization using the steepest descent method of the MMFF94s force field [24], which was used to eliminate poor atomic contacts and geometries.

### 2.3. Molecular Fingerprint Similarity Calculation

Molecular fingerprinting, as a method of characterizing chemical structures, is widely used in clustering or recursive partitioning and similarity searching [25]. The molecular fingerprint similarity of the dietary flavonoids was calculated using the Open-Source Cheminformatics Software RDKit (version 2021.09, http://www.rdkit.org/ (accessed on 12 June 2022)). Additionally, Simplified Molecular-Input Line-Entry System (SMILES) formats and topological fingerprints for each dietary flavonoid compound were obtained using the RDKit software. Finally, hierarchical clustering of dietary flavonoids was performed using the single-link algorithm of the Python scikit-learn library (version 0.23.2, https://scikit-learn.org.cn/ (accessed on 20 July 2022)) [26].

### 2.4. MLR-QSAR Modeling

The 2D and 3D molecular descriptors for each dietary flavonoid compound were obtained using the PaDEL-Descriptor software package (version 2.21, http://www.yapcwsoft.com/dd/padeldescriptor/ (accessed on 3 August 2022)) [27]. PaDEL descriptors contain 1875 descriptors (1444 1D and 2D descriptors, and 431 3D descriptors). The 2D molecular descriptors represent structural information that can be calculated from the 2D structure of the molecule, such as the number of benzene rings, the number of hydrogen bond donors, etc. The 3D molecular descriptors represent structural information that must be obtained from the 3D representation of the molecule (e.g., the solvent-accessible and surface area of the structure with a positive partial charge). Additionally, parameters such as the 3D autocorrelation charged part surface area, gravity index, length and width, moment of inertia, Petitjean shape index, RDF, and WHIM were described. Then, blank columns and columns with all zeros were removed, and correlation analysis was performed to remove the high covariance parameter (*p* > 0.9). Finally, genetic algorithms were used to further optimize these parameters, and the pIC_50_ values of the flavonoids were iteratively optimized to build a multiple linear regression (MLR)-QSAR model.

### 2.5. Validation of the MLR-QSAR Model

The prediction ability and stability of the MLR-QSAR model were evaluated by using the parameters Q^2^ (leave-one-out cross-validation), R^2^ fitting, adjusted R^2^, RSS, PRESS, SDEC, SDEP, MSE, and MAE of partial least squares analysis. The formula definitions of these parameters can be found in the Supplementary Materials (Table S1).

### 2.6. Natural Product Screening

Firstly, molecular fingerprint similarity clustering analysis was performed on the target molecules, and the MolNatSim tool based on the COCONUT database was used to match the similarity of each flavonoid in this study to establish a clustering library of the target compounds. To rapidly locate the most similar natural product molecules and perform conditional screening, pre-optimized molecular similarity prediction models (molecular ECFP4 fingerprint and mini-batch k-means algorithm) are required [28]. In addition, the MolNatSim tool also provides visual analysis operations. Finally, the generated MLR-QSAR model and ADMET prediction analysis were used to make predictions for the possibility of the screened natural product molecules.

### 2.7. Molecular Docking and Molecular Dynamics Simulation

AutoDock Vina was used for molecular docking to obtain complexes in potentially optimal poses [29]. The 3D conformational isomers of the flavonoids were downloaded from PubChem and optimized using RDKit software based on the MMFF94s force field. The crystal structure of α-glucosidase from *Saccharomyces cerevisiae* is still not available., so we used the homology model reported by Jia et al. [21]. The structure was taken from the Protein Data Bank (PDB ID: 3A4A). ORCA 5.0.3 was used to calculate the structural optimization vibration and single-point energy, and the calculation level used for vibration and optimization was r2scan-3c [30]. The single-point energy used was RI-B3LYP-D3(BJ)/def2-TZVP [31], and the RESP charge was fitted using Multiwfn [32]. The small molecule Topol was obtained by fitting the bond and angle parameters based on the Hessian matrix using Sobtop [33]. Molecular dynamics (MD) simulations were used to explain conformational changes in the complex ligand–receptor binding interactions and stability [34]. All MD simulations in the current work used the GROMACS 19.5 package (https://manual.gromacs.org/ (accessed on 20 August 2022)) [35]. The docking structure models of flavonoids and α-glucosidase proteins were subjected to 50 ns MD simulations in solution with AMBER14SB [36] and the general AMBER force field (GAFF), respectively. The water box adopted the TIP3P water model with a minimum distance of 1.0 nm between the solute atoms and the edge of the periodic box. The system removed overlapping water molecules, and appropriate amounts of Na^+^/Cl^−^ ions were added to neutralize the system. The energy minimization was conducted in two steps by gauge ensemble (NVT) and isothermal isobaric ensemble. After MD simulations, the root-mean-square deviation (RMSD) and root-mean-square fluctuation (RMSF) of the complexes were analyzed using the GROMACS package. Unless otherwise stated, other parameters can be found in our previous work [37].

### 2.8. Combined Free Energy Calculation

Molecular mechanics/generalized Born surface area (MMGBSA) was used to calculate the binding free energy of the enzyme protein receptor and ligand small molecule complexes [38]. This algorithm can be used to resolve complex interactions between complex molecules by decomposing and calculating the components that make up the binding free energy. This method extracts the architecture from the MD simulation trajectory of the complex at certain time intervals and calculates the average binding free energy. As a scoring function, the MMGBSA calculation method is widely used in drug design. MMGBSA was used in this study to obtain binding free energies for the design of α-glucosidase inhibitors.

## 3. Results and Discussion

### 3.1. Molecular Fingerprint Similarity Analysis

**Figure 1:**
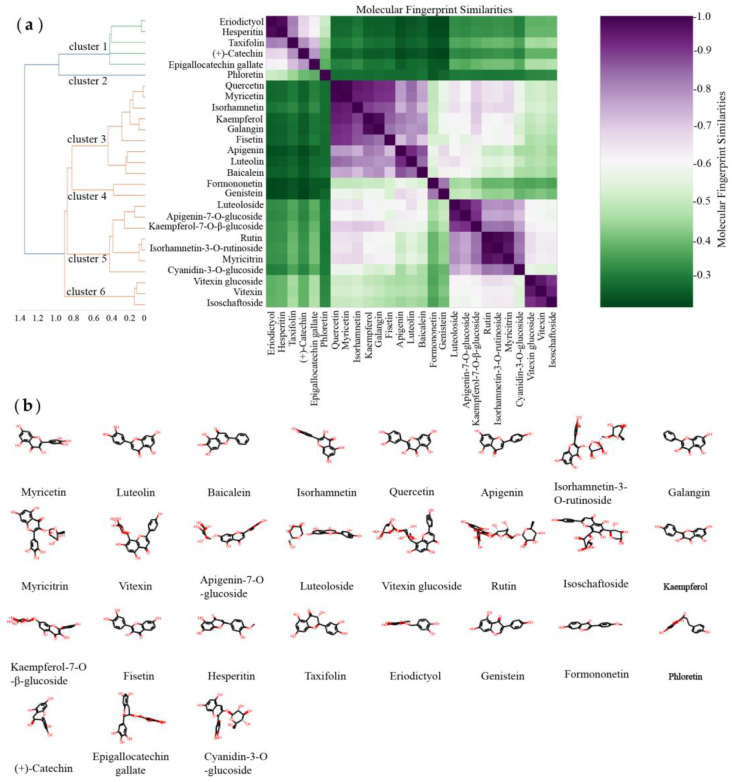
(**a**) Hierarchical clustering of molecular fingerprint similarities of dietary flavonoid compounds. (**b**) Structures of 27 dietary flavonoid compounds.

Molecular fingerprints are special qualitative molecular descriptors originally designed for similarity searching, comparison, and clustering of molecules, and they are widely used in drug discovery and virtual screening [15]. The molecular similarity method was developed based on the principle of molecular similarity (i.e., structurally similar molecules should exhibit the same or similar biological activity) to search for compounds with similar characteristics to known ligands. Therefore, to reveal the difference in the α-glucosidase-inhibitory activity of dietary flavonoids, fingerprint similarity analysis was performed on the selected dietary flavonoid compounds (Figure 1).

The hierarchical clustering results of the molecular fingerprint similarity of the selected dietary flavonoid compounds are shown in Figure 1. The more purple the color of the heatmap, the higher the molecular fingerprint similarity (MFS). Flavonoids are structurally composed of two benzene rings (A and B rings) with phenolic hydroxyl groups linked by three central carbon atoms [39]. According to the hierarchical clustering results of molecular fingerprint similarity, these dietary flavonoids can be classified into six categories according to the differences in the three-carbon-atom structure connecting the A ring and the B ring, such as whether the ring is formed, oxidized, and/or substituted. Flavanones (e.g., hesperitin, taxifolin, and eriodictyol) and (+)-catechin, with the largest IC_50_ values, can be clustered as Cluster 1 (mean MFS score > 0.6). Among them, epigallocatechin gallate and (+)-catechin both belong to flavan-3-ols and are therefore clustered in Cluster 1. Since phloretin belongs to the dihydrochalcones, it was separately aggregated as Cluster 2. Flavonoids that do not contain O-glucose groups (e.g., myricetin, luteolin, baicalein, isorhamnetin, quercetin, apigenin, galangin, kaempferol, fisetin) were clustered into Cluster 3 (mean MFS score > 0.7). Isoflavones (e.g., genistein, formononetin) were clustered into one category as Cluster 4, which had a higher paired MFS score (mean MFS score > 0.8). Anthocyanins (e.g., cyanidin-3-*O*-glucoside) and flavonoids containing the O-glucose group (e.g., isorhamnetin-3-*O*-rutinoside, myricitrin, apigenin-7-*O*-glucoside, luteoloside, rutin, kaempferol-7-*O*-β-glucoside) were clustered into Cluster 5 (mean MFS score > 0.75). Among the flavonoids, vitexin, vitexin glucoside, and isoschaftoside, with more similar side-chain linkages, were clustered into one category as Cluster 6, which had the highest paired MFS scores (mean MFS score > 0.9). Overall, the hierarchical clustering results showed that the molecular fingerprint similarity and structures of the flavonoids were in good agreement. 

### 3.2. MLR-QSAR Analysis

**Figure 2:**
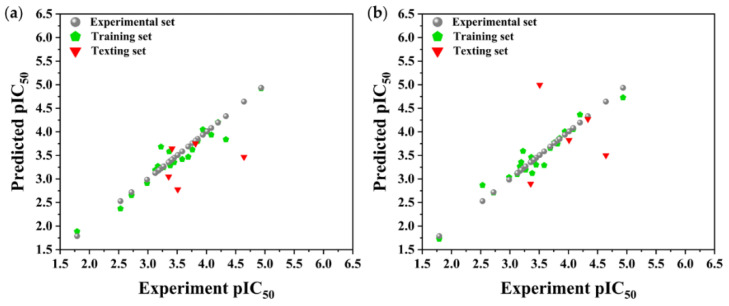
MLR-QSAR linear regression models. (**a**) 2D-QSAR model of dietary flavonoids. (**b**) 3D-QSAR model of dietary flavonoids.

QSAR modeling is a method for establishing quantitative relationships between the structural or physicochemical parameters of compounds and their biological activities using mathematical calculation and statistical analysis based on MLR. Predicting the biological activity of unknown compounds based on the MLR-QSAR model can significantly reduce the cost of target compound screening and design. We used the PaDEL-Descriptor software package to generate 2D and 3D descriptors to fully characterize the structure of the dietary flavonoids, analyzing the autocorrelation of the descriptors while excluding highly linear descriptors. Finally, a genetic algorithm was used to further optimize the descriptors, and the pIC_50_ values were iteratively modeled to construct the MLR-QSAR [40]. The results in Figure 2 show that all of the points are located near the diagonal line, indicating a good correlation between the experimental inhibition rate (pIC_50_) and the predicted inhibition rate (pIC_50pre_), suggesting that the model is very predictable (Table 1). In the 2D-MLR-QSAR model, the R^2^ fitting was 0.9273, the R^2^ adjusted was 0.9046, and the Q^2^_LOO_ was 0.8861. In the 3D-MLR-QSAR model, the R^2^ fitting, R^2^ adjusted, and Q^2^_LOO_ were 0.9336, 0.9129, and 0.8991, respectively (Table 2). These results indicate that the developed model has a good fit and could adequately predict the inhibitory effects of dietary flavonoid compounds on α-glucosidase.

The RSS, PRESS, SDEC, SDEP, MSE, and MAE parameters were calculated to further evaluate the robustness of the model, and the results are shown in Table 2. The RSS of the 2D and 3D QSAR models was 0.6906 and 0.5679, the PRESS was 1.0829 and 0.8633, the SDEC was 0.1772 and 0.1607, the SDEP was 0.2219 and 0.1981, the MSE was 0.1636 and 0.2545, and the MAE was 0.3138 and 0.349, respectively. These results show that this model has good robustness and predictive capability, and that the predictive performance of 2D-MLR-QSAR is better than that of 3D-MLR-QSAR.

Next, the relationships between the structural properties and pIC_50_ values of the dietary flavonoids were further analyzed based on key descriptors. In the MLR equation of the 2D-QSAR model, MIC1, ATS4v, AATS7m, CIC3, and minssCH2 were identified as key descriptors affecting the inhibitory activity of dietary flavonoids on α-glucosidase (Table 2). The maximum coefficient of CIC3 was 3.06615, and the coefficient of AATS7m was 0.05492, contributing positively to the model. Meanwhile, the coefficients of MIC1, ATS4v, and minssCH2 were −0.15599, −0.00011, and −3.00189, respectively, contributing negatively to the model. ATS4v is a molecular descriptor for characterizing the topology of compounds based on the intrinsic states of atoms [41]. AATS7m corresponds to the length of the branch on the R group in the molecular structure of dietary flavonoids [42]. CIC3 is the complementary information content index (third-order neighborhood symmetry). MIC1 is a molecular descriptor that considers steric effects in molecule–receptor interactions based on proximity and edge multiplicity [43]. Unlike the MLR equation of the 2D-QSAR model, LOBMAX, RDF35i, TDB10i, TDB9i, and TDB6m in the MLR equation of the 3D-QSAR model were considered to be the key descriptors affecting the inhibitory activity of dietary flavonoids on α-glucosidase (Table 2). Among them, TDB10i, TDB9i, and TDB6m were considered to be based on molecular 3D topological distance autocorrelation [43]. Based on the coefficients of these parameters listed in Table 2, LOBMAX, TDB10i, and TDB6m were 0.49135, 0.00505, and 0.01155, respectively, contributing positively to the model, while the other two parameters (RDF35i and TDB9i) contributed negatively to the model. RDF35i corresponds to the radial distribution function—055/weighted by the first ionization potential, which describes the molecular radial distribution function [44].

**Figure 3:**
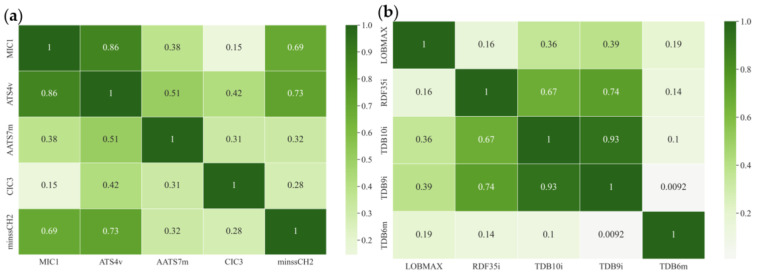
Optimal autocorrelation analysis in MLR−QSAR models: (**a**) 2D−QSAR model of dietary flavonoids. (**b**) 3D−QSAR model of dietary flavonoids.

Based on Pearson’s correlation coefficients, autocorrelation analysis of the two descriptors used to train the MLR-QSAR model was conducted. Figure 3a,b show the optimal autocorrelation plots of the descriptors for the 2D-MLR- and 3D-MLR-QSAR models of the flavonoids, respectively. The results showed that the pairwise correlation of most of the key descriptors was less than 0.8, indicating that the correlation between these key descriptors was not strong. It follows that the constructed model can be used to quantify the inhibitory activity of dietary flavonoids on α-glucosidase. Since the 2D-MLR-QSAR model had better predictive performance, it was used for prediction and screening in subsequent study. 

### 3.3. Discovery of Natural α-Glucosidase Inhibitors and ADMET Analysis

To better apply the model to predict the potential natural α-glucosidase inhibitors, we used the COCONUT-based MolNatSim tool (which contains over 400,000 natural products) to match the 27 natural dietary flavonoid molecules modeled in this paper. Refer to our previous work for an introduction to the MolNatSim Tool [45]. Natural product clustering libraries with five different clustering degrees were used to enrich each molecule. Firstly, the Morgan fingerprint similarity between the molecules in each molecular arrowhead and the target molecule was calculated using RDKit, and molecules with a similarity greater than 80% were retained. Secondly, a total of 1716 natural molecules were enriched by merging 27 molecular arrowheads. Finally, 1198 natural molecules were obtained by deduplication according to SMILE and number.

Drug similarity is a key consideration when selecting compounds in the early stages of drug discovery. Applying quantitative estimation of drug-likeness (QED) to the molecularly targeted druggability assessment problem facilitates drug discovery by prioritizing a large number of published biologically active compounds [46]. Therefore, QED and other screening methods should be used to accelerate the discovery of α-glucosidase-inhibitory activity of natural dietary flavonoid molecules based on similarity. We chose a QED value of 0.5 and MWs of 650 as the threshold to reduce the number of molecules from 852 to 82 and removed the elements not contained in the molecules from the modeling. The results are shown in Table S2, and 78 natural product molecules were obtained, including the 3 molecules in the modeling. The prediction of pIC_50pre_ was carried out using the 2D-MLR-QSAR model, and the results are shown in Table S2. Finally, five natural products were found to meet the requirements that pIC_50pre_ be larger than the pIC_50_ of the 27 natural dietary flavonoid molecules. These were 2-(3,5-dihydroxyphenyl)-5,7-dihydroxy-4*H*-chromen-4-one, norartocarpetin, 2-(2,5-dihydroxyphenyl)-5,7-dihydroxy-4*H*-chromen-4-one, 2-(3,4-dihydroxyphenyl)-5-hydroxy-4*H*-chromen-4-one, and morelosin. The quantitative conformational relationship predictions showed that their IC_50pre_ values were 8.98, 31.95, 78.57, 87.87, and 94.14 µM, respectively.

To assess bioavailability and avoid drug interactions based on the proposed natural compounds, the prediction of ADMET properties was conducted to reduce potential problems in later clinical trials. For this purpose, the pharmacokinetics of these novel natural product α-glucosidase inhibitors were considered using SwissADME web server [47] (http://www.swissadme.ch (accessed on 17 August 2022)) and PreADMET web server [48] (https://preadmet.bmdrc.kr (accessed on 18 August 2022)) predictions. ADMET parameters were calculated for the five natural products, and the results are shown in Table 3. Solubility is an important parameter in ADMET prediction to evaluate the possibility of intestinal absorption and blood distribution. Three of these natural products—2-(3,5-dihydroxyphenyl)-5,7-dihydroxy-4*H*-chromen-4-one, norartocarpetin, and 2-(2,5-dihydroxyphenyl)-5,7-dihydroxy-4*H*-chromen-4-one—had good solubility, and all five natural products may have good bioavailability and gastrointestinal absorption. In addition, the blood–brain barrier (BBB) is the main interface separating the central nervous system from the blood circulation [49]. Our results showed that these five natural products have non-permeable BBB ability. Pgp is an important transporter protein responsible for the excretion of many harmful substances from cells, and naturally also for the removal of many drugs from cells [50]. Our results showed that these five natural products are not Pgp substrates, indicating they cannot reduce the debilitating effects of drug efficacy. Fortunately, our candidate compounds 2-(3,5-dihydroxyphenyl)-5,7-dihydroxy-4*H*-chromen-4-one and norartocarpetin are not expected to show toxicity to cancerous rat and mouse cells. As shown in Table S3, based on drug similarity predictions, the natural compounds conformed to Lipinski’s rule, Veber’s rule, and Egan’s rule, indicating that the compounds can be easily synthesized [51]. Thus, these studies provide favorable support for our model to predict the outcomes of natural α-glucosidase inhibitors.

### 3.4. Molecular Docking Analysis

**Figure 4:**
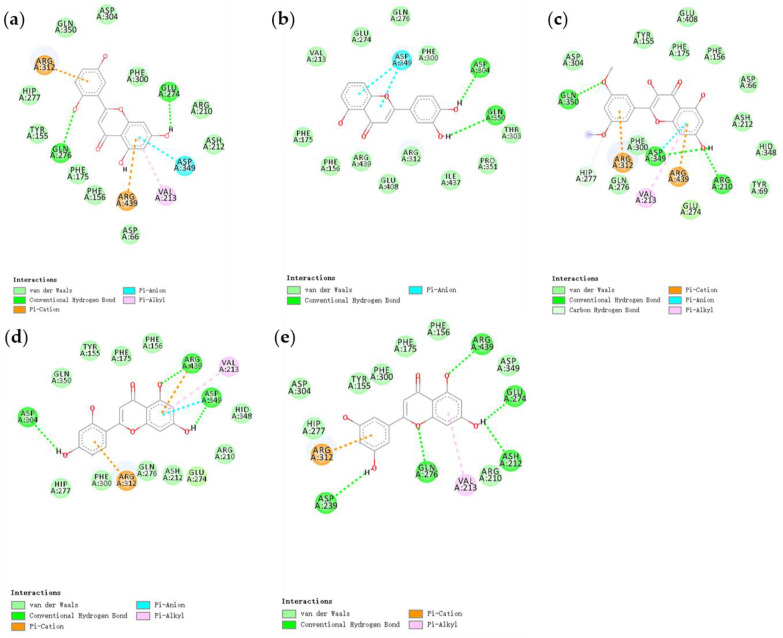
Docking interactions between five novel natural products and α-glucosidase 3A4A: (**a**) 2-(2,5-dihydroxyphenyl)-5,7-dihydroxy-4H-chromen-4-one; (**b**) 2-(3,4-dihydroxyphenyl)-5-hydroxy-4H-chromen-4-one; (**c**) morelosin; (**d**) norartocarpetin; (**e**) 2-(3,5-dihydroxyphenyl)-5,7-dihydroxy-4H-chromen-4-one.

We used AutoDock Vina for molecular docking to explore the possible binding modes of five natural compounds with α-glucosidase. The interaction results were visualized and analyzed using Discovery Studio 2016 software. The docking results showed many interactions between the five natural flavonoid compounds and α-glucosidase. Figure 4a–e show the interaction of the ligands (2-(2,5-dihydroxyphenyl)-5,7-dihydroxy-4*H*-chromen-4-one, 2-(3,4-dihydroxyphenyl)-5-hydroxy-4H-chromen-4-one, morelosin, norartocarpetin, and 2-(3,5-dihydroxyphenyl)-5,7-dihydroxy-4H-chromen-4-one, respectively) with the amino acid residues of α-glucosidase. As shown in Figure 4, the compound 2-(2,5-dihydroxyphenyl)-5,7-dihydroxy-4*H*-chromen-4-one interacts with the active site of α-glucosidase, forming conventional hydrogen bond interactions between two amino acid residues (Gln276 and Glu274), and forming π-anion and π-cation interactions between Asp349 and Arg312, as well as π-alkyl interactions with residues of Val 213. It has been reported that gallic acid catechins exhibit strong inhibition of α-glucosidase in a non-competitive manner by reacting with Arg 312 and others [52]. For the compound 2-(3,4-dihydroxyphenyl)-5-hydroxy-4*H*-chromen-4-one, there are two main interactions with the α-glucosidase receptor: the π-anion interaction with residues of Asp349, and the formation of hydrogen bonds between Asp304 and Gln350. Morelosin and 2-(2,5-dihydroxyphenyl)-5,7-dihydroxy-4*H*-chromen-4-one similarly interact with α-glucosidase receptors in a variety of ways, with conventional hydrogen bond interactions forming between Gln350, Asp349, and Arg210, and π-cation and π-anion interactions with Arg312 and Arg439. The compounds norartocarpetin and morelosin formed similar interactions with the α-glucosidase receptor, forming conventional hydrogen bond interactions with Asp349, Asp304, and Arg439, π-cation interactions with Arg312, and π-alkyl interactions with residues of Val213. Another study found that Arg439 and Val213 were important for the catalytic reaction of α-glucosidase [53]. 2-(3,5-Dihydroxyphenyl)-5,7-dihydroxy-4*H*-chromen-4-one mainly formed hydrogen bond interactions with α-glucosidase receptors with Arg439, Glu274, Ash212, Gln276, and Asp239. In addition, π-cation and π-alkyl interactions were formed with Arg312 and Val213, respectively. In a previous study [21], acarbose was used as a positive control to react with amino acid residues of α-glucosidase, including Glu274, Asp349, Arg439, and Arg 312. These were used to stabilize the enzyme–ligand complex in the catalytic reaction, so these amino acid residues affected the catalytic action of α-glucosidase. We found that five natural flavonoid compounds could form hydrogen bonds with multiple residues of α-glucosidase. The hydrogen bonding interaction force is thought to play a key role in stabilizing the enzyme–ligand complex to perform the catalytic reaction, which depends mainly on the number of hydrogen bonds [54]. 2-(3,5-Dihydroxyphenyl)-5,7-dihydroxy-4*H*-chromen-4-one, morelosin, and norartocarpetin have more hydrogen bonding interactions with the α-glucosidase receptor and, therefore, are able to form more stable enzyme–ligand complexes.

### 3.5. Molecular Dynamics Simulation Analysis

**Figure 5:**
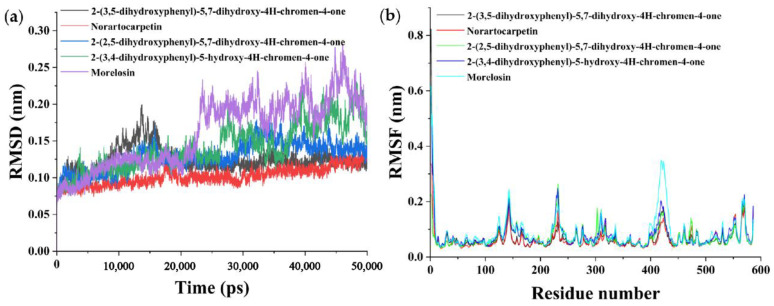
Molecular dynamics simulation (50 ns) results of five novel natural products complexed with α-glucosidase: (**a**) Root-mean-square deviation (RMSD, nm). (**b**) Root-mean-square fluctuation (RMSF, nm).

The dynamic stability and binding energy of the complexes were further analyzed by molecular dynamics (MD) simulations. RMSD was used to determine the average deviation of the complex conformation from the original conformation at a given time and to assess whether the complex system had reached a steady state [55]. As shown in Figure 5a, both 2-(3,5-dihydroxyphenyl)-5,7-dihydroxy-4*H*-chromen-4-one and 2-(2,5-dihydroxyphenyl)-5,7-dihydroxy-4*H*-chromen-4-one systems reached equilibrium at 20,000 ps, and their RMSD values remained stable at 0.12 and 0.14 nm, respectively. Both 2-(3,4-dihydroxyphenyl)-5-hydroxy-4*H*-chromen-4-one and morelosin showed a rise in their RMSD values in the first 25,000 ps of the simulation and then became more stable at 45,000 ps while reaching equilibrium, and their RMSD values remained stable at 0.20 and 0.18 nm, respectively. The norartocarpetin system reached equilibrium faster, within 15,000 ps, and was stable at an average value of 0.10 nm. Moreover, norartocarpetin was the least fluctuating system overall among the five systems. In summary, the five systems converged and balanced within 50,000 ps of simulation, with 2-(3,5-dihydroxyphenyl)-5,7-dihydroxy-4H-chromen-4-one, 2-(2,5-dihydroxyphenyl)-5,7-dihydroxy-4H-chromen-4-one, and norartocarpetin showing the best stability.

RMSF can be used to characterize the stability of a composite system by evaluating the fluctuation of conformational change from the original conformation [55]. Figure 5b shows the RMSF values of the five natural product–α-glucosidase complex molecules as a function of residue number. In the active center, GLU274, GLN276, ASP304, GLN350, and ARG439 are the key amino acid residues. The binding of the five natural products to the α-glucosidase receptor leads to the increased flexibility of residues (212–239), (274–304), and (407–439) in the critical region. This suggests that novel natural flavonoid compounds may inhibit α-glucosidase activity by interacting with key residues affecting the activity pocket. As shown in Figure 5b, the mean values of RMSF for all residues of the five natural products were 0.07, 0.06, 0.07, 0.08, and 0.09 nm, respectively. Thus, the natural products 2-(3,5-dihydroxyphenyl)-5,7-dihydroxy-4*H*-chromen-4-one, norartocarpetin, and 2-(2,5-dihydroxyphenyl)-5,7-dihydroxy-4H-chromen-4-one have lower flexibility (or higher rigidity), except for a limited number of regions. The RMSF provided information on the local structural mobility of the proteins during the MD simulations. Among the five environments, the natural products 2-(3,5-dihydroxyphenyl)-5,7-dihydroxy-4*H*-chromen-4-one, norartocarpetin, and 2-(2,5-dihydroxyphenyl)-5,7-dihydroxy-4*H*-chromen-4-one showed lower residue fluctuations, indicating that these three natural products have fewer conformational changes and higher stability after binding to α-glucosidase.

### 3.6. Combining Free Energy Calculations by the MMGBSA Method

To quantitatively characterize the effects of the interactions between the five novel natural products and α-glucosidase, we calculated the binding free energy of the two products using the MMGBSA algorithm. After choosing 40–50 ns and taking conformations at certain intervals, the MD of the complex systems was simulated by the MM-GBSA algorithm. As shown in Table 4, all of the complexes showed good binding free energies; the simulated values of free energy of binding (∆G_bind_) for the five complexes were −23.87, −37.78, −27.81, −22.53, and −16.56 kcal/mol, respectively. The negative values of the free energy of binding indicate that the process of binding is spontaneous and can reach a steady state [56]. Among all types of interactions, the van der Waals interaction energy term (ΔE_vdw_) and the electrostatic interaction energy term (∆E_ele_) contribute the most to the average binding free energy, providing the main driving force for the binding of novel natural products to α-glucosidases. The non-polar solvation energy term (∆G_non-pol_) also acts as a key force to maintain the stability of the system. ΔG_gas_ includes van der Waals energy (ΔE_vdw_), electrostatic energy (ΔE_ele_), and non-polar solvation energy (∆G_non-pol_) [56]. However, the polar solvation energy (∆G_pol_) exhibited an unfavorable energetic contribution, inhibiting the spontaneous binding of the natural product to α-glucosidase, but was mostly counteracted by the molecular electrostatic interaction energy under vacuum conditions. Notably, in terms of the total binding free energy, the natural product inhibitors norartocarpetin, 2-(3,5-dihydroxyphenyl)-5,7-dihydroxy-4*H*-chromen-4-one, and 2-(2,5-dihydroxyphenyl)-5,7-dihydroxy-4H-chromen-4-one can form more stable complexes with α-glucosidase.

## 4. Conclusions

In this study, the molecular fingerprint similarity clustering analysis was carried out on the target molecules, and then the results of the MLR-QSAR model construction showed a good correlation between the experimental value (pIC_50_) and the predicted value (pIC_50pre_), meaning that the model can be used to predict the properties of new and potential dietary flavonoid compounds. Finally, the MolNatSim tool, based on the COCONUT database, was used to build the clustering library of the target compounds. After a series of screenings, five natural products (2-(3,5-dihydroxyphenyl)-5,7-dihydroxy-4*H*-chromen-4-one, norartocarpetin, 2-(2,5-dihydroxyphenyl)-5,7-dihydroxy-4*H*-chromen-4-one, 2-(3,4-dihydroxyphenyl)-5-hydroxy-4*H*-chromen-4-one, and morelosin) were found to have good α-glucosidase-inhibitory activity. The results of ADMET assessed properties such as the bioavailability and drug similarity of natural products. Molecular docking analysis revealed the interactions of the candidate novel natural flavonoid compounds with the amino acid residues of α-glucosidase. MD simulations and MMGBSA further validated the stability of these novel natural compounds bound to α-glucosidase. In particular, the compounds 2-(3,5-dihydroxyphenyl)-5,7-dihydroxy-4*H*-chromen-4-one, norartocarpetin, and 2-(2,5-dihydroxyphenyl)-5,7-dihydroxy-4*H*-chromen-4-one (predicted pIC_50pre_ values of 8.98, 31.95, and 78.57 µM, respectively) can be considered potent novel α-glucosidase inhibitors.

## Figures and Tables

**Table 1:** Experimental and 2D- or 3D-MLR-QSAR predicted pIC_50_ values for α-glucosidase-inhibitory activity of 27 dietary flavonoids.

2D-MLR-QSAR					3D-MLR-QSAR				
PubChem CID	Name	pIC_50_ *	pIC_50pre_ #	Residue	PubChem CID	Name	pIC_50_	pIC_50pre_	Residue
5280443	Apigenin	3.224	3.684	−0.46	5280445	Luteolin	4.082	4.054	0.028
5281605	Baicalein	3.586	3.42	0.166	5280863	Kaempferol	3.814	3.746	0.068
5281616	Galangin	3.381	3.284	0.097	5280443	Apigenin	3.224	3.593	−0.37
5281654	Isorhamnetin	3.17	3.271	−0.102	5280343	Quercetin	3.939	4.008	−0.07
5280445	Luteolin	4.082	3.938	0.144	5281654	Isorhamnetin	3.17	3.264	−0.095
5281672	Myricetin	4.934	4.92	0.014	5281672	Myricetin	4.934	4.729	0.206
5280863	Kaempferol	3.814	3.751	0.064	5280704	Apigenin-7-*O*-glucoside	4.642	3.502	1.14
5280343	Quercetin	3.939	4.05	−0.111	5281605	Baicalein	3.586	3.29	0.296
5280704	Apigenin-7-*O*-glucoside	4.642	3.468	1.174	5281614	Fisetin	4.334	4.271	0.063
72281	Hesperitin	2.72	2.654	0.067	440735	Eriodictyol	2.986	3.035	−0.049
5280441	Vitexin	3.448	3.353	0.095	5281616	Galangin	3.381	3.122	0.259
5281614	Fisetin	4.334	3.839	0.494	439533	Taxifolin	2.531	2.869	−0.338
439533	Taxifolin	2.531	2.371	0.16	72281	Hesperitin	2.72	2.708	0.012
440735	Eriodictyol	2.986	2.911	0.075	5280378	Formononetin	3.126	3.101	0.025
4788	Phloretin	3.85	3.806	0.045	5280637	Luteoloside	3.365	3.463	−0.098
9064	(+)-Catechin	1.789	1.888	−0.098	5280961	Genistein	3.192	3.357	−0.164
5280637	Luteoloside	3.365	3.582	−0.217	5280441	Vitexin	3.448	3.302	0.147
5280378	Formononetin	3.126	3.189	−0.063	3084995	Isoschaftoside	3.411	3.366	0.045
5280961	Genistein	3.192	3.219	−0.027	172648475	Kaempferol-7-*O*-β-glucoside	3.689	3.654	0.035
172648475	Kaempferol-7-*O*-β-glucoside	3.689	3.47	0.219	9064	(+)-Catechin	1.789	1.729	0.06
5281673	Myricetin	3.51	2.776	0.734	5281673	Myricetin	3.51	4.997	−1.487
441667	Cyanidin-3-*O*-glucoside	4.197	4.204	−0.006	5280805	Rutin	3.761	3.771	−0.01
65064	Epigallocatechin Gallate	4.011	4.009	0.002	5481663	Isorhamnetin-3-*O*-rutinoside	3.268	3.196	0.072
3084995	Isoschaftoside	3.411	3.646	−0.234	56776173	Vitexin-4′-*O*-glucoside	3.354	2.898	0.456
5280805	Rutin	3.761	3.623	0.138	4788	Phloretin	3.85	3.865	−0.015
56776173	Vitexin-4′-*O*-glucoside	3.354	3.049	0.306	65064	Epigallocatechin Gallate	4.011	3.826	0.185
5481663	Isorhamnetin-3-*O*-rutinoside	3.268	3.245	0.023	441667	Cyanidin-3-*O*-glucoside	4.197	4.365	−0.168

* pIC_50_ = experimental value. # pIC_50pre_ = predicted pIC_50_ value.

**Table 2:** MLR-QSAR models (2D- and 3D-descriptor optimized) of 27 dietary flavonoids and cross-validation results.

Parameters	2D-MLR-QSAR	3D-MLR-QSAR
Q^2^_LOO_	0.8861	0.8991
R^2^ fitting	0.9273	0.9336
R^2^ adjusted	0.9046	0.9129
RSS	0.6906	0.5679
PRESS	1.0829	0.8633
SDEC	0.1772	0.1607
SDEP	0.2219	0.1981
MSE	0.1636	0.2545
MAE	0.3138	0.349
MLR	pIC_50_ = 4.12506 + (−0.15599) × MIC1 + (−0.00011) × ATS4v + (0.05492) × AATS7m + (3.06615) × CIC3 + (−3.00189) × minssCH2	pIC_50_ = 8.97844 + (0.49135) LOBMAX + (−0.03043) × RDF35i + (0.00505) × TDB10i + (−0.01383) × TDB9i + (0.01155) × TDB6m

**Table 3:** The 2D-MLR-QSAR prediction and ADMET analysis of potential natural α-glucosidase inhibitors.

Compounds	Molecular Structure	IC_50pre_ # (μM)	Water Solubility	Bioavailability Score	GI Absorption	BBB Permeant	Pgp Substrate	log Kp (cm/s)	Toxicity	
									Carcino_Mouse	Carcino_Rat
2-(3,5-Dihydroxyphenyl)-5,7-dihydroxy-4*H*-chromen-4-one	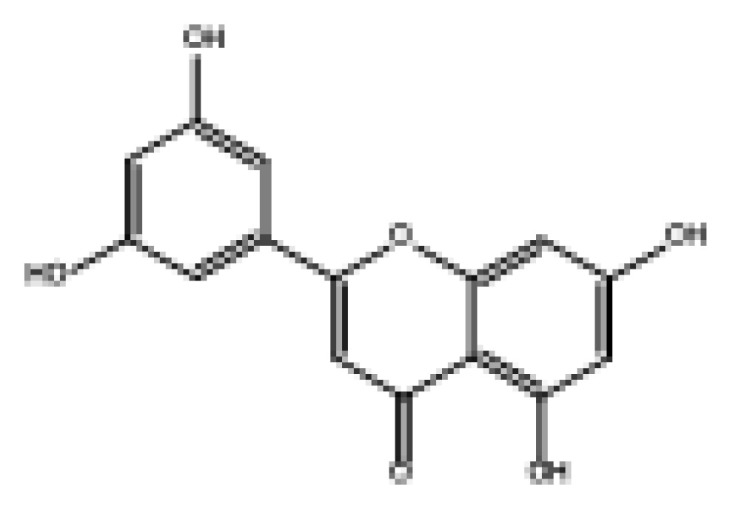	8.98	Soluble	0.55	High	No	No	−6.05	Negative	Negative
Norartocarpetin	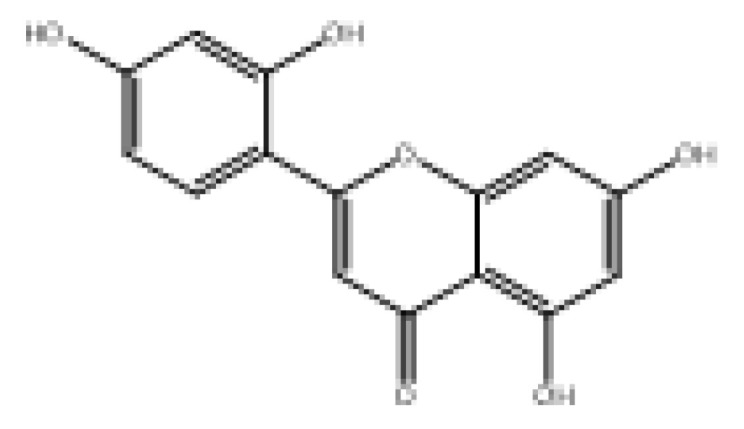	31.95	Soluble	0.55	High	No	No	−6.16	Negative	Negative
2-(2,5-Dihydroxyphenyl)-5,7-dihydroxy-4*H*-chromen-4-one	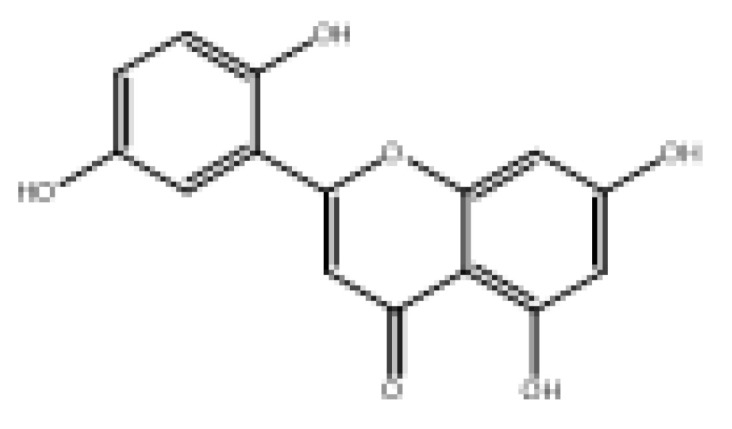	78.57	Soluble	0.55	High	No	No	−6.16	Negative	Positive
2-(3,4-Dihydroxyphenyl)-5-hydroxy-4*H*-chromen-4-one	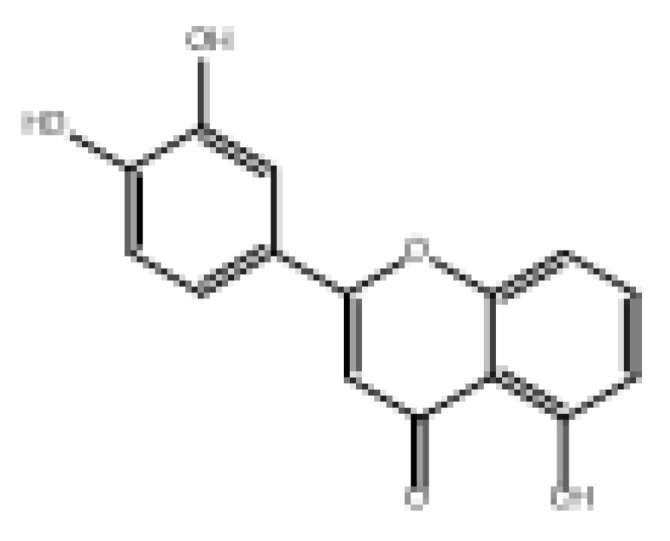	87.87	Moderately soluble	0.55	High	No	No	−5.6	Negative	Positive
Morelosin	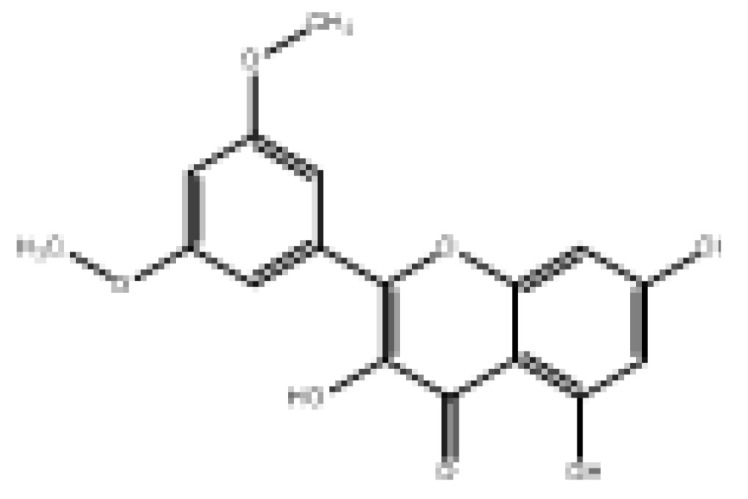	94.14	Moderately soluble	0.55	High	No	No	−6.76	Negative	Positive

# pIC_50pre_ = predicted pIC_50_ values.

**Table 4:** Binding free energy of complex formation between α-glucosidase and its inhibitors.

Energy (kJ/mol)	2-(3,5-Dihydroxyphenyl)-5,7-Dihydroxy-4*H*-Chromen-4-one	Norartocarpetin	2-(2,5-Dihydroxyphenyl)-5,7-Dihydroxy-4*H*-Chromen-4-one	2-(3,4-Dihydroxyphenyl)-5-Hydroxy-4*H*-Chromen-4-one	Morelosin
ΔE_vdw_	−23.24 ± 1.95	−30.29 ± 2.98	−26.31 ± 4.52	−35.28 ± 2.77	−29.34 ± 2.45
∆E_ele_	−38.62 ± 5.45	−53.66 ± 5.59	−46.07 ± 9.18	−15.2 ± 10.68	−7.1 ± 3.49
∆G_pol_	41.82 ± 3.12	51.32 ± 3.38	48.7 ± 8.00	32.38 ± 7.23	23.99 ± 3.64
∆G_non-pol_	−3.84 ± 0.27	−5.15 ± 0.13	−4.13 ± 0.34	−4.43 ± 0.15	−4.11 ± 0.23
∆G_gas_	−61.86 ± 5.44	83.95 ± 4.93	−72.38 ± 10.14	−50.48 ± 9.98	−36.43 ± 4.19
∆G_sol_	37.98 ± 3.01	46.17 ± 3.36	44.57 ± 7.72	27.95 ± 7.25	19.88 ± 3.52
∆G_bind_	−23.87 ± 3.18	−37.78 ± 3.28	−27.81 ± 3.45	−22.53 ± 3.40	−16.56 ± 2.55

Note: ΔE_vdw_, the van der Waals interaction energy term; ∆E_ele_, the electrostatic interaction energy term; ∆G_pol_, the polar solvation energy term; ∆G_non-pol_, The non-polar solvation energy term; ∆G_gas_, the gas phase free energy term; ∆G_sol_, the solvation free energy term; ∆G_bind_, the free energy of binding.

## Data Availability

Data are contained within the article or the Supplementary Materials.

## Supplementary Materials

The following supporting information can be downloaded at: https://www.mdpi.com/article/10.3390/foods11244046/s1, Table S1: Equations presented in the research paper; Table S2 2D-MLR-QSAR prediction of potential natural α-glucosidase inhibitors; Table S3. Drug similarity prediction of natural products based on Lipinski, Veber and Egan rules.
