# Supplementary material for: Construction of an MLR-QSAR Model Based on Dietary Flavonoids and Screening of Natural α-Glucosidase Inhibitors
Source: Foods. 2022 Dec 14;11(24):4046. doi: 10.3390/foods11244046 (PMC9778400; doi:10.3390/foods11244046)
Supplement: Supplementary file 1 [file foods-11-04046-s001.zip › foods-2067380-supplementary.pdf]

Supplementary File

Table S1 Equations presented in the research paper

Sl. No.	Equation
**1**	$R^2 = 1 - \frac{SS_{res}}{SS_{tot}} = 1 - \frac{\sum_i (y_i - f_i)^2}{\sum_i (y_i - \bar{y}_i)^2}$
**2**	Adjusted $R^2 = 1 - \frac{PRESS/df_e}{SS_{tot}/df_t}$
**3**	$RSS = \sum_i (y_i - f_i)^2$
**4**	$PRESS = \sum_{i=1}^{n} \left(y_i - f_{i/i}\right)^2$
**5**	$SDEC = \sqrt{\frac{RSS}{n}}$
**6**	$SDEP = \sqrt{\frac{PRESS}{n}}$
**7**	$MAE(X, h) = \frac{1}{m} \sum_{i=1}^{m} \lvert h(x_i) - y_i \rvert$
**8**	$MSE = \frac{1}{m} \sum_{i=1}^{m} (y_i - \hat{y}_i)^2$

Note: y_i , f_i , $\bar{y}$ are the experimental, predicted, and average values, respectively. SS_{res} is the sum of squares of residuals, also called the residual sum of squares. SS_{tot} is the total sum of squares. df_e is the degree of freedom of the underlying overall error variance estimate n-p-1. df_t is the degree of freedom n-1 of the overall variance estimate of the dependent variable.

Table S2 2D-MLR-QSAR prediction of potential natural α-glucosidase inhibitors

name	QED	MW	ALOGP	HBA	HBD	ROTB	PSA	AROM	ALERTS	smile
5281605	0.5911	270.24	2.5768	5	3	1	90.9	3	1	O=c1cc(-c2ccccc2)oc2cc(O)c(O)c(O)c12
5281654	0.5721	316.265	2.291	7	4	2	120.36	3	0	COc1cc(-c2oc3cc(O)cc(O)c3c(=O)c2O)ccc1O
5280445	0.5106	286.239	2.2824	6	4	1	111.13	3	1	O=c1cc(-c2ccc(O)c(O)c2)oc2cc(O)cc(O)c12
CNP0401811	0.6769	330.292	2.594	7	3	3	109.36	3	0	COc1cc(-c2oc3cc(O)cc(OC)c3c(=O)c2O)ccc1O
CNP0229011	0.6769	330.292	2.594	7	3	3	109.36	3	0	COc1ccc(-c2oc3cc(O)cc(O)c3c(=O)c2O)cc1OC
CNP0237570	0.6769	330.292	2.594	7	3	3	109.36	3	0	COc1cc(O)c2c(=O)c(O)c(-c3ccc(O)c(OC)c3)oc2c1
CNP0137967	0.5721	316.265	2.291	7	4	2	120.36	3	0	COc1ccc(-c2oc3cc(O)cc(O)c3c(=O)c2O)cc1O
CNP0304960	0.6769	330.292	2.594	7	3	3	109.36	3	0	COc1cc(-c2oc3cc(O)cc(O)c3c(=O)c2OC)ccc1O
CNP0070352	0.6723	314.293	2.8938	6	3	2	100.13	3	0	COc1cc(-c2oc3cc(O)cc(O)c3c(=O)c2C)ccc1O
CNP0085812	0.6567	315.257	1.659	7	3	2	123.19	3	0	COc1cc(-c2oc3cc([O-])cc(O)c3c(=O)c2O)ccc1O
CNP0251015	0.6318	270.24	2.5768	5	3	1	90.9	3	0	O=c1cc(-c2ccc(O)cc2)oc2cc(O)cc(O)c12
CNP0290456	0.5106	286.239	2.2824	6	4	1	111.13	3	1	O=c1cc(-c2ccc(O)cc2)oc2cc(O)c(O)c(O)c12
CNP0311055	0.6388	284.267	2.8852	5	3	1	90.9	3	0	Cc1cc(-c2cc(=O)c3c(O)cc(O)cc3o2)ccc1O

CNP0303945	0.626	285.231	1.6504	6	3	1	113.96	3	0	O=c1cc(-c2ccc([O-])c(O)c2)oc2cc(O)cc(O)c12
CNP0226932	0.7107	268.268	3.1796	4	2	1	70.67	3	0	Cc1c(O)cc2oc(-c3ccccc3)cc(=O)c2c1O
CNP0305733	0.7107	268.268	3.1796	4	2	1	70.67	3	0	Cc1cc(O)c2c(=O)cc(-c3ccc(O)cc3)oc2c1
CNP0149125	0.6549	254.241	2.8712	4	2	1	70.67	3	1	O=c1cc(-c2ccccc2)oc2ccc(O)c(O)c12
CNP0394762	0.6289	300.266	2.5854	6	3	2	100.13	3	1	COc1cc(O)c2c(=O)cc(-c3ccc(O)c(O)c3)oc2c1
CNP0292269	0.5459	286.239	2.2824	6	4	1	111.13	3	0	O=c1c(O)c(-c2ccc(O)cc2)oc2cc(O)cc(O)c12
CNP0102699	0.514	300.266	2.5908	6	4	1	111.13	3	1	Cc1c(-c2ccc(O)c(O)c2)oc2cc(O)cc(O)c2c1=O
CNP0356234	0.7107	268.268	3.1796	4	2	1	70.67	3	0	Cc1c(-c2ccccc2)oc2cc(O)cc(O)c2c1=O
CNP0076419	0.7037	269.232	1.9448	5	2	1	93.73	3	0	O=c1c(O)c(-c2ccccc2)oc2cc([O-])cc(O)c12
CNP0167158	0.5352	316.265	2.291	7	4	2	120.36	3	1	COc1cc(O)cc2oc(-c3ccc(O)c(O)c3)c(O)c(=O)c12
CNP0145919	0.756	284.267	2.8798	5	2	2	79.9	3	0	COc1cc(O)c2c(=O)cc(-c3ccc(O)cc3)oc2c1
CNP0065787	0.6649	268.268	3.1796	4	2	1	70.67	3	1	Cc1cc2oc(-c3ccccc3)cc(=O)c2c(O)c1O
CNP0399551	0.5459	286.239	2.2824	6	4	1	111.13	3	0	O=c1c(O)c(-c2ccccc2O)oc2cc(O)cc(O)c12
CNP0291526	0.6723	300.266	2.5854	6	3	2	100.13	3	0	COc1ccc(-c2oc3cc(O)cc(O)c3c(=O)c2O)cc1
CNP0116129	0.756	284.267	2.8798	5	2	2	79.9	3	0	COc1cc(O)c2c(=O)c(O)c(-c3ccccc3)oc2c1
CNP0236338	0.6318	270.24	2.5768	5	3	1	90.9	3	0	O=c1c(O)c(-c2ccccc2)oc2cc(O)cc(O)c12
CNP0242521	0.7506	344.319	2.897	7	2	4	98.36	3	0	COc1cc(O)c2c(=O)c(O)c(-c3ccc(OC)c(OC)c3)oc2c1

CNP0185958	0.6496	360.318	2.6026	8	3	4	118.59	3	0	COc1cc(-c2oc3cc(OC)c(OC)c(O)c3c(=O)c2O)ccc1O
CNP0112748	0.5683	346.291	2.2996	8	4	3	129.59	3	0	COc1cc(-c2oc3cc(O)c(OC)c(O)c3c(=O)c2O)ccc1O
CNP0194512	0.7001	254.241	2.8712	4	2	1	70.67	3	0	O=c1cc(-c2ccc(O)cc2)oc2cccc(O)c12
CNP0269044	0.5459	286.239	2.2824	6	4	1	111.13	3	0	O=c1cc(-c2cc(O)cc(O)c2)oc2cc(O)cc(O)c12
CNP0376829	0.5459	286.239	2.2824	6	4	1	111.13	3	0	O=c1cc(-c2ccc(O)cc2O)oc2cc(O)cc(O)c12
CNP0287080	0.6318	270.24	2.5768	5	3	1	90.9	3	0	O=c1cc(-c2cccc(O)c2)oc2cc(O)cc(O)c12
CNP0110361	0.7506	344.319	2.897	7	2	4	98.36	3	0	COc1ccc(-c2oc3cc(O)cc(OC)c3c(=O)c2O)cc1OC
CNP0246782	0.6723	300.266	2.5854	6	3	2	100.13	3	0	COc1ccc(-c2cc(=O)c3c(O)cc(O)cc3o2)cc1O
CNP0223782	0.6723	300.266	2.5854	6	3	2	100.13	3	0	COc1cc(-c2cc(=O)c3c(O)cc(O)cc3o2)ccc1O
CNP0268505	0.6289	300.266	2.5854	6	3	2	100.13	3	1	COc1cc(O)cc2oc(-c3ccc(O)c(O)c3)cc(=O)c12
CNP0315728	0.5494	300.266	2.5908	6	4	1	111.13	3	0	Cc1cc(-c2oc3cc(O)cc(O)c3c(=O)c2O)ccc1O
CNP0280624	0.7001	254.241	2.8712	4	2	1	70.67	3	0	O=c1cc(-c2ccccc2)oc2cc(O)cc(O)c12
CNP0194512	0.5911	270.24	2.5768	5	3	1	90.9	3	1	O=c1cc(-c2ccc(O)c(O)c2)oc2cccc(O)c12
CNP0205924	0.5106	286.239	2.2824	6	4	1	111.13	3	1	O=c1cc(-c2ccccc2O)oc2cc(O)c(O)c(O)c12
CNP0241556	0.5106	286.239	2.2824	6	4	1	111.13	3	1	O=c1c(O)c(-c2ccccc2)oc2cc(O)c(O)c(O)c12
CNP0192878	0.756	284.267	2.8798	5	2	2	79.9	3	0	COc1c(O)cc2oc(-c3ccccc3)cc(=O)c2c1O

CNP0214218	0.6769	330.292	2.594	7	3	3	109.36	3	0	COc1ccc(-c2oc3cc(O)cc(OC)c3c(=O)c2O)cc1O
CNP0292859	0.6723	300.266	2.5854	6	3	2	100.13	3	0	COc1cc(-c2oc3cc(O)ccc3c(=O)c2O)ccc1O
CNP0105978	0.6769	330.292	2.594	7	3	3	109.36	3	0	COc1cc(OC)cc(-c2oc3cc(O)cc(O)c3c(=O)c2O)c1
CNP0279025	0.5683	346.291	2.2996	8	4	3	129.59	3	0	COc1cc(-c2oc3cc(O)cc(O)c3c(=O)c2O)cc(OC)c1O
CNP0115195	0.5352	316.265	2.291	7	4	2	120.36	3	1	COc1c(-c2ccc(O)c(O)c2)oc2cc(O)cc(O)c2c1=O
CNP0269649	0.5683	346.291	2.2996	8	4	3	129.59	3	0	COc1cc(-c2oc3cc(O)cc(O)c3c(=O)c2O)c(OC)cc1O
CNP0241196	0.67	344.319	2.9024	7	3	3	109.36	3	0	COc1cc(-c2oc3cc(O)cc(O)c3c(=O)c2O)cc(C)c1OC
CNP0076076	0.626	285.231	1.6504	6	3	1	113.96	3	0	O=c1c(O)c(-c2ccc([O-])cc2)oc2cc(O)cc(O)c12
CNP0219017	0.5459	286.239	2.2824	6	4	1	111.13	3	0	O=c1c(O)c(-c2cccc(O)c2)oc2cc(O)cc(O)c12
CNP0145877	0.756	284.267	2.8798	5	2	2	79.9	3	0	COc1cc(O)cc2oc(-c3ccccc3)c(O)c(=O)c12
CNP0199169	0.756	284.267	2.8798	5	2	2	79.9	3	0	COc1c(-c2ccccc2)oc2cc(O)cc(O)c2c1=O
CNP0124637	0.7506	344.319	2.897	7	2	4	98.36	3	0	COc1cc(-c2oc3cc(O)cc(OC)c3c(=O)c2OC)ccc1O
CNP0298914	0.7506	344.319	2.897	7	2	4	98.36	3	0	COc1ccc(-c2oc3cc(O)cc(O)c3c(=O)c2OC)cc1OC
CNP0293671	0.6769	330.292	2.594	7	3	3	109.36	3	0	COc1cc(O)c2c(=O)c(O)c(-c3ccc(OC)c(O)c3)oc2c1
CNP0391817	0.5106	286.239	2.2824	6	4	1	111.13	3	1	O=c1cc(-c2cc(O)ccc2O)oc2cc(O)cc(O)c12

CNP0325061	0.7072	284.267	2.8798	5	2	2	79.9	3	1	COc1cc2oc(-c3ccccc3)cc(=O)c2c(O)c1O
CNP0228332	0.6289	300.266	2.5854	6	3	2	100.13	3	1	COc1ccc(-c2cc(=O)c3c(O)c(O)c(O)cc3o2)cc1
CNP0304229	0.573	278.263	3.9118	4	1	1	63.58	4	0	O=c1cc(-c2ccccc2)oc2cc3ccoc3c(O)c12
CNP0266609	0.6723	300.266	2.5854	6	3	2	100.13	3	0	COc1ccccc1-c1oc2cc(O)cc(O)c2c(=O)c1O
CNP0113555	0.5721	316.265	2.291	7	4	2	120.36	3	0	COc1cc(O)ccc1-c1oc2cc(O)cc(O)c2c(=O)c1O
CNP0330206	0.5352	316.265	2.291	7	4	2	120.36	3	1	COc1cc(O)c2c(=O)c(O)c(-c3ccc(O)c(O)c3)oc2c1
CNP0179264	0.708	238.242	3.1656	3	1	1	50.44	3	0	O=c1cc(-c2ccccc2)oc2cccc(O)c12
CNP0266076	0.756	284.267	2.8798	5	2	2	79.9	3	0	COc1cc(O)cc2oc(-c3ccc(O)cc3)cc(=O)c12
CNP0184323	0.7725	314.293	2.8884	6	2	3	89.13	3	0	COc1ccc(-c2cc(=O)c3c(O)cc(O)cc3o2)cc1OC
CNP0229255	0.7175	298.25	2.5999	6	2	1	89.13	3	0	O=c1cc(-c2ccc3c(c2)OCO3)oc2cc(O)cc(O)c12
CNP0120950	0.5683	346.291	2.2996	8	4	3	129.59	3	0	COc1cc(-c2oc3cc(O)cc(O)c3c(=O)c2O)cc(O)c1OC
CNP0300748	0.6723	300.266	2.5854	6	3	2	100.13	3	0	COc1cc(O)cc2oc(-c3ccc(O)cc3)c(O)c(=O)c12
CNP0425896	0.6496	360.318	2.6026	8	3	4	118.59	3	0	COc1cc(OC)c(-c2oc3cc(O)cc(O)c3c(=O)c2O)cc1OC
CNP0048956	0.5798	342.347	3.4809	6	4	3	111.13	3	0	CC(C)Cc1cc(-c2oc3cc(O)cc(O)c3c(=O)c2O)ccc1O
CNP0392086	0.7506	344.319	2.897	7	2	4	98.36	3	0	COc1cc(O)c2c(=O)c(OC)c(-c3ccc(O)c(OC)c3)oc2c1
CNP0248340	0.756	284.267	2.8798	5	2	2	79.9	3	0	COc1ccc(-c2cc(=O)c3c(O)cc(O)cc3o2)cc1

CNP0322103	0.6496	360.318	2.6026	8	3	4	118.59	3	0	COc1cc(-c2oc3cc(O)cc(O)c3c(=O)c2O)cc(OC)c1OC

Table S3. Drug similarity prediction of natural products based on Lipinski, Veber and Egan rules.

Compounds	Lipinski rule	Ghose rule	Veber rule	Egan rule	Muegge rule
CNP0269044	Yes	Yes	Yes	Yes	Yes
CNP0376829	Yes	Yes	Yes	Yes	Yes
CNP0391817	Yes	Yes	Yes	Yes	Yes
CNP0194512	Yes	Yes	Yes	Yes	Yes
CNP0105978	Yes	Yes	Yes	Yes	Yes
